# Cross-dataset annotation harmonization for cell-type hierarchy construction

**DOI:** 10.1093/bioinformatics/btag506

**Published:** 2026-07-09

**Authors:** Tianhong Zhou, Yixin Chen, Yingtao Zhu, Jinmeng Jia, Xuegong Zhang, Lei Wei

**Affiliations:** MOE Key Laboratory of Bioinformatics and Bioinformatics Division of BNRIST, Department of Automation, Tsinghua University, Beijing 100084, China; College of Biomedical Engineering, Fudan University, Shanghai 200438, China; School of Medicine, Tsinghua Medicine, Tsinghua University, Beijing 100084, China; MOE Key Laboratory of Bioinformatics and Bioinformatics Division of BNRIST, Department of Automation, Tsinghua University, Beijing 100084, China; MOE Key Laboratory of Bioinformatics and Bioinformatics Division of BNRIST, Department of Automation, Tsinghua University, Beijing 100084, China; Center for Synthetic and Systems Biology, School of Life Sciences and School of Medicine, Tsinghua University, Beijing 100084, China; MOE Key Laboratory of Bioinformatics and Bioinformatics Division of BNRIST, Department of Automation, Tsinghua University, Beijing 100084, China

## Abstract

**Motivation:**

Single-cell transcriptomic datasets annotate cell types with diverse schemes and varying resolution. This poses challenges in building unified hierarchical cell-type structures and hinders integration of large-scale datasets. To address this, several computational methods have been developed to harmonize cell type annotations across datasets and build data-driven hierarchies of cell types.

**Results:**

Here, we benchmarked three state-of-the-art methods: scHPL, treeArches, and CellHint. We evaluated these methods across five simulated scenarios and five real-world scenarios across cell types and organs. To assess harmonization results, we designed three metrics, Annotation Harmonization F1-score (AH-F1), Tree Edit Distance Similarity and Parent–Children Branches Similarity, comparing the constructed cell-type hierarchies and the knowledge-based ones. Based on the benchmarking results, we found that methods performed well in simulated scenarios but still have room for improvement in complex real-world data. Thus, we developed OTHarmonizer, a tool based on partial optimal transport (OT) for cell-type harmonization and hierarchy construction. OTHarmonizer excels in accurately capturing equivalent and hierarchical relationships between cell types, offering a more effective approach for the cell-type hierarchy construction across datasets.

**Availability and implementation:**

The simulated and real-world datasets in the benchmark are available on https://figshare.com/articles/dataset/OTHarmonizer/28243205. The source codes for the benchmark and OTHarmonizer are available online on GitHub at https://github.com/Duck-Boss/OTHarmonizer.

## 1 Introduction

Single-cell sequencing technologies, with its exceptional precision and depth, enable researchers to investigate cell types at the molecular level (Stoeckius *et al.* 2017). This capability has paved the way for constructing comprehensive cell atlases (Regev *et al.* 2017, Rozenblatt-Rosen *et al.* 2017, HuBMAP Consortium 2019, Chen *et al.* 2022) as well as hierarchical reference trees of cell types within the human body (Börner *et al.* 2021, Osumi-Sutherland *et al.* 2021, Chen *et al.* 2022, Domcke and Shendure 2023). However, the inconsistencies in naming schemes and resolutions of annotation across datasets pose significant challenges to data integration and the establishment of unified cell-type hierarchies. Harmonizing cell-type annotations has become a crucial and foundational step toward integrating large-scale single-cell datasets and building cell atlases (Osumi-Sutherland *et al.* 2021, Sikkema *et al.* 2023, Johnston *et al.* 2024).

An intuitive solution of harmonizing cell-type annotations is to map the original annotations onto a pre-constructed cell-type hierarchy. Frameworks like the Cell Ontology (Diehl *et al.* 2016), the Anatomical Structures, Cell Types, and Biomarkers (ASCT+B) framework from Human BioMolecular Atlas Program and our unified hierarchical cell annotation framework in human Ensemble Cell Atlas (hECA) provides viable options based on knowledge and literature mining (HuBMAP Consortium^ 2019^, Börner *et al.* 2021, Chen *et al.* 2022). However, manual alignment is not only labor-intensive but also requires specialized expertise to interpret the original annotations and cell distributions (Zeisel *et al.* 2018). Additionally, the mapping results are heavily influenced by the quality of original annotations and annotation granularity.

Another solution is to re-annotate cells in each dataset. However, both manual annotation and automatic annotation methods face significant challenges. Manual annotation relies on marker gene databases such as CellMarker 2.0, PanglaoDB, and ASCT+B, which often ignore the hierarchical relationships between cell types (Franzén *et al.* 2019, Zhang *et al.* 2019a, Börner *et al.* 2021, Hu *et al.* 2023). Additionally, literature-based markers may lack robustness across datasets. Automatic annotation tools, such as scmap and CHETAH, are susceptible to batch effects, and reference-to-query tools, such as Seurat and scANVI (Butler *et al.* 2018, Kiselev *et al.* 2018, de Kanter *et al.* 2019, Stuart *et al.* 2019, Xu *et al.* 2021), are computationally intensive in pairwise alignment and may ignore hierarchical relationship between cell types. More importantly, all these approaches rely on a high-quality reference dataset or a comprehensive reference cell-type hierarchy. This creates a paradox, as such reference datasets or hierarchies are often the intended outcomes of cell atlas construction and are unavailable during the early stages of the process.

**Figure 1:**
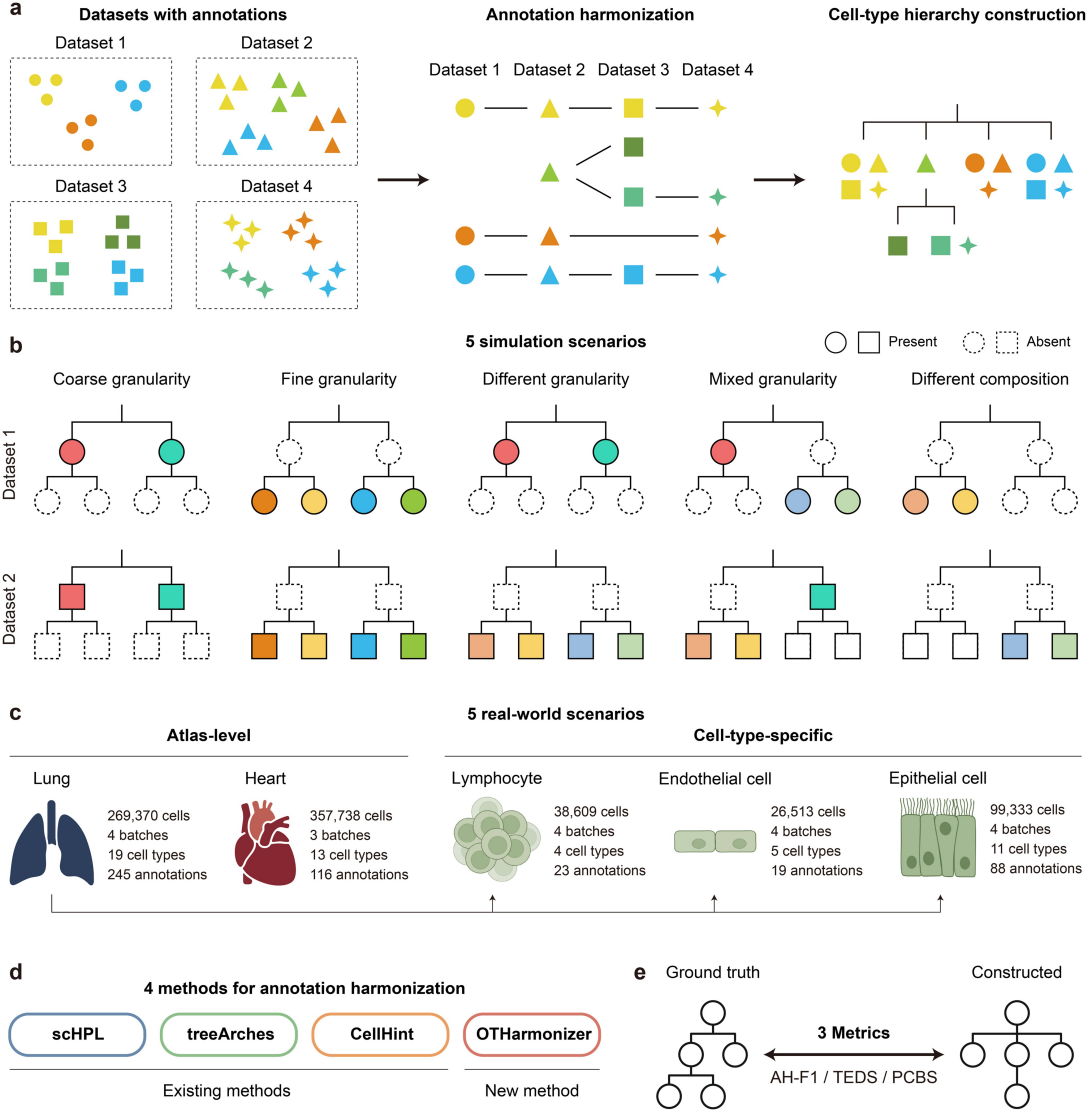
Task definition and benchmark design of cross-dataset annotation harmonization and cell-type hierarchy construction. (a) The standardize pipeline of the existed methods for annotation harmonization. These methods first learned the relationship between the original annotation labels across the input datasets, and then constructed a cell-type hierarchy with the result of the first step. (b) Illustration of the design of the five simulated scenarios to evaluate the performance of harmonization tools. In the cell-type hierarchy, colors of the node represent the cell types and the subtypes. The solid line represents the presence of the annotations in the dataset, while the dashed line indicates its absence. (c) Overview of the human lung and heart datasets, including lung subsets: lymphocytes, endothelial cells, and epithelial cells. The figure shows dataset size, cell types, and annotations, highlighting the complexity of annotation and data harmonization challenges. (d) Four methods for annotation harmonization: scHPL, treeArches, CellHint, and OTHarmonizer. (e) Evaluation of ground truth versus constructed cell-type hierarchies based on AH-F1, TEDS, and PCBS metrics.

In recent years, several automatic methods provided data-driven approaches to leverage both data similarity and information from original annotations to identify relationships between cell types and subtypes. These methods typically divided the task into two steps: cell-type annotation harmonization and cell hierarchy construction (Fig. 1a). For instance, Michielsen *et al.* (2021) proposed scHPL, a cross-dataset alignment method based on hierarchical progressive learning. It constructs a hierarchical classifier using multiple flat classifiers, leverages confusion matrices to determine relationships between annotations, and subsequently builds a hierarchical tree for alignment. However, batch effects obscure biological signals, adversely impacting classification performance and alignment accuracy in this work. To address this, Michielsen’s team further released treeArches, which mitigates batch effects using scArches before performing annotation alignment to enhance accuracy (Lotfollahi *et al.* 2022, Michielsen *et al.* 2023). Besides, Xu *et al.* (2023) developed CellHint, a method that utilizes predictive clustering trees to align multiple scRNA-seq datasets effectively. By assessing cell similarity and coordinating annotations, CellHint can capture potential biological hierarchies among cell types, offering a solution for harmonizing annotations and revealing structural relationships.

Despite these advancements, a systematic benchmark for evaluating performance and guiding method selection is still lacking. Common evaluation metrics for flatten classifiers, such as accuracy and F1-score, failed to capture the hierarchical nature of cell types, particularly the differing importance of higher- and lower-level cell types within the hierarchy. This underscores the necessity for specialized metrics that better reflect hierarchical structural similarity. Although scHPL introduced some metrics for assessing hierarchical structures, its performance, as well as that of other methods, remains largely unexamined and lacks comparative evaluation.

To address these limitations, a framework that can robustly capture cross-dataset relationships and flexibly handle unmatched or heterogeneous cell types is needed. OT provides a natural solution because it aligns cell distributions rather than relying only on local neighborhoods or classifier-based assignment. This is particularly suitable for annotation harmonization, where annotations across datasets are rarely in perfect one-to-one correspondence and more often show partial overlap, containment relationships, or dataset-specific populations. Full matching may force all cells to be assigned to a counterpart, whereas partial OT allows only the shared part of two distributions to be transported and leaves unmatched mass unforced. The resulting transport plan offers a quantitative and biologically interpretable description of how cell populations correspond across datasets. With entropic regularization enabling efficient large-scale computation, partial OT forms a principled foundation for annotation harmonization and hierarchical structure inference.

Building on this motivation, we developed OTHarmonizer, a partial OT-based method for cross-dataset annotation harmonization and cell-type hierarchy construction, and designed a benchmark to systematically evaluate this task. Our benchmark includes five simulated scenarios generated using the simulation tool Symsim (Zhang *et al.* 2019b), three relatively simple scenarios involving cell-type-specific real-world data, and two complex, atlas-scale scenarios involving organ-specific real-world data from Human Lung Cell Atlas (HLCA) and hECA heart data (Chen *et al.* 2022, Sikkema *et al.* 2023). We developed three evaluation metrics tailored for this task: Annotation Harmonization F1-score (AH-F1) to measure annotation consistency, and Tree Edit Distance Similarity (TEDS) and Parent–Children Branches Similarity (PCBS) to evaluate hierarchical structure accuracy. Existing methods treeArches, scHPL, and CellHint, showed notable limitations in the benchmark, particularly in complex real-world scenarios, with issues like poor harmonization accuracy, sensitivity to batch effects, and long computation time. To address these gaps, we further developed OTHarmonizer, leveraging partial optimal transport (OT) to effectively align cell types and construct hierarchical relationships (Bonneel *et al.* 2011, Cuturi 2013). OTHarmonizer demonstrates superior accuracy and efficiency in the benchmark, offering a new tool for cell-type annotation harmonization and hierarchy construction.

## 2 Materials and methods

### 2.1 Datasets for benchmarking

For the simulated scenarios, we used the R package Symsim (V0.0.0.9000) to generate scRNA-seq data containing cell subtype hierarchy information. The data includes five simulated scenarios, each consisting of three batches. For the coarse granularity and fine granularity scenario, each dataset contains 5500 cells and 10 cell types. The coarse granularity scenario includes 18 annotations, and the fine granularity scenario includes 30 annotations. For the different granularity and mixed granularity scenario, each dataset contains 9000 cells and 9 cell types. The different granularity scenario includes 19 annotations, and the mixed granularity scenario includes 20 annotations. The different composition scenario includes 5000 cells, 9 cell types, and 17 annotations.

The reference hierarchy tree is provided in Fig. 3a, with each cell type containing more than 500 cells. A batch effect with an intensity of 0.65 was applied to the data when requiring batch effect influence.

For the real-world scenarios, we used publicly available datasets. The HLCA data can be downloaded from https://data.humancellatlas.org/hca-bio-networks/lung/atlases/lung-v1-0. The hECA data can be downloaded from https://ngdc.cncb.ac.cn/omix/release/OMIX008091. Lymphoid, Endothelial, and Epithelial cells were extracted based on the original annotation. Cell annotations were harmonized by concatenating the dataset name and original fine-grained labels. Standard Scanpy (Wolf *et al.* 2018) preprocessing was applied: library-size normalization (normalize_total, target sum 1 × 10^4^), log-transformation, HVG selection with dataset-aware handling, PCA, neighborhood graph construction, and UMAP embedding.

### 2.2 Construction of the ground truth hierarchy

For simulated data, the ground truth is inherently available, as the data is generated based on a pre-defined hierarchical tree. For real-world datasets, we constructed the ground-truth hierarchies with particular caution to avoid any bias that might inadvertently favor our method. Wherever possible, ground truths were directly adopted from authoritative reference atlases (e.g. hECA). For HLCA, whose published hierarchy is relatively coarse, we introduced only minimal refinements supported by validated marker information (e.g. CellMarker 2.0) to ensure biological correctness without imposing any method-dependent structure. As an example, we show the refinement process for the HLCA lymphocyte scenario in Fig. S9, available as supplementary data at *Bioinformatics* online, including the revised tree and the supporting marker-gene evidence. Each node in the final hierarchy corresponds to a cell-type annotation present in the datasets, yielding a biologically reliable and method-agnostic ground-truth tree for evaluation.

### 2.3 Metrics in the benchmark

#### 2.3.1 Annotation harmonization F1-score

AH-F1 evaluates how accurately a predicted hierarchy preserves biological relationships between all pairs of annotated cell types, compared with the ground-truth hierarchy. For any two annotations of one tree A and B, their relationship belongs to one of three mutually exclusive groups in a given hierarchy:

- Parent–Child: One annotation is an ancestor of the other (e.g. T cell → CD4 T cell).
- Equal: A and B refer to the same biological concept and occupy the same node in the hierarchy.
- Non-relation: A and B reside on different branches and share no equality or ancestor–descendant relationship.

For each relationship group ($G_{1}$: parent–child, $G_{2}$: equal, $G_{3}$: non-relation), we calculate the Precision, Recall, and F1 score. For Set of relationships in the query tree $Q_{i}$ and in the reference tree $R_{i}$:


$${\mathrm{Precision}}_{i}=\frac{\left|Q_{i}\cap R_{i}\right|}{\left|Q_{i}\right|}$$

where $\left|Q_{i}\cap R_{i}\right|$ is the size of the intersection of the two sets and $\left|Q_{i}\right|$ is the size of $Q_{i}$.


Recalli=|Qi∩Ri||Ri|F1i={2⋅Precisioni⋅RecalliPrecisioni+Recalli, if Precisioni+Recalli>00, if Precisioni+Recalli=0

We calculate the AH-F1 score by averaging the F1 scores of all three groups:


$$\text{AH}-F1=\frac{{F1}_{1}+{F1}_{2}+{F1}_{3}}{3}$$

#### 2.3.2 Tree edit distance similarity

Tree Edit Distance (TED) is a widely used method for measuring the structural differences between two trees (Zhang and Shasha 1989). The core idea of TED is to calculate the minimum cost required to transform one tree $T_{1}$ into another tree $T_{2}$ using a series of edit operations. These operations include:

- Node Insertion: Adding a new node to the tree.
- Node Deletion: Removing a node from the tree.
- Node Substitution: Replacing one node with another.

Each operation is associated with a cost, $\mathrm{cost}(e_{i})$, where different operations may have different costs. The TED is defined as the minimum total cost of the required edit operations:


$$\mathrm{TED}(T_{1},T_{2})=\min\sum_{i=1}^{k}\;\mathrm{cost}(e_{i})$$

where $e_{i}$ represents the i-th edit operation, and k is the total number of operations needed.

Since TED measures only the absolute differences between two trees, it does not directly provide a standardized similarity metric. To address this, we normalize TED to define the similarity TEDS. First, we calculate the maximum possible edit distance between the two trees:


$${\mathrm{TED}}_{\max}(T_{1},T_{2})=\mathrm{NodeNum}(T_{1})+\mathrm{NodeNum}(T_{2})$$

Here NodeNum(T) represents the total number of nodes in tree T. The normalized TEDS is then defined as:


$$\mathrm{TEDS}(T_{1},T_{2})=1-\frac{\mathrm{TED}(T_{1},T_{2})}{{\mathrm{TED}}_{\max}(T_{1},T_{2})}$$

TEDS ranges from [0,1]. A TEDS value of 1 indicates that the two trees are identical, while a value of 0 indicates complete dissimilarity. TEDS provides an effective global similarity metric that captures structural and positional consistency between trees.

#### 2.3.3 Parent–children branches similarity

In a tree structure, each parent node p and its child nodes $c_{1},c_{2},\ldots ,c_{k}$ form a parent–children set:


$$S=\{p,c_{1},c_{2},\ldots ,c_{k}\}$$

PCBS measures the similarity between such parent–children sets in two trees. Suppose we have two trees, $T_{1}$ and $T_{2}$ and their parent–children sets:$\;S_{a}\in T_{1}$, which represent the parent–children set in tree $T_{1}$, and $S_{b}\in T_{2}$, which represent parent–children set in tree $T_{2}$. The matching score between $S_{a}$ and $S_{b}$ is defined as:


$$\mathrm{Score}(S_{a},S_{b})=\frac{\left|S_{a}\cap S_{b}\right|}{\left|S_{a}\cup S_{b}\right|}$$

For every parent-children set $S_{a}\in T_{1}$, we find the most similar parent-children set $S_{b}\in T_{2}$ with the highest matching score. The overall PCBS is calculated as:


$$\mathrm{PCBS}(T_{1},T_{2})=\frac{\sum_{S_{a}\in T_{1}}\;\underset{S_{b}\in T_{2}}{\max}\;(\mathrm{Score}(S_{a},S_{b}))+\sum_{S_{b}\in T_{2}}\;\underset{S_{a}\in T_{1}}{\max(}\;\mathrm{Score}(S_{b},S_{a}))}{\left|T_{1}|+|T_{2}\right|}$$

where $\left|T_{1}\right|$ is the total number of parent–children sets in $T_{1}$.

PCBS ranges from [0,1]. A PCBS value of 1 indicates that every parent–children set in $T_{1}$ finds a perfectly matching set in $T_{2}$, while a value of 0 indicates no matching. PCBS provides a fine-grained evaluation of local relationships between nodes, making it particularly useful for capturing detailed structural differences.

#### 2.3.4 Average silhouette width for batch

Batch ASW is used to quantify how well batches are mixed in an integrated latent space after batch correction. It is adapted from the classic Silhouette coefficient, with batch labels serving as the grouping variable. For a given cell i, the batch silhouette width $s_{i}$ is defined as:


$$s_{i}=\frac{b_{i}-a_{i}}{\max(a_{i},b_{i})}$$

where $a_{i}$ denotes the average distance from cell i to all other cells within the same batch, and $b_{i}$ represents the lowest average distance from cell i to any other batch. Intuitively, $a_{i}$ measures within-batch compactness, whereas $b_{i}$ reflects the degree to which cell i is close to cells from other batches.

Batch ASW is computed as the mean silhouette width across all cells. Higher Batch ASW values indicate that cells are closer to cells of the same batch and farther from other batches, reflecting stronger batch separation. Conversely, lower Batch ASW implies that batches are more intermixed, typically indicating more effective batch correction and reduction of batch-driven clustering. The Batch ASW score ranges from 0 to 1.

### 2.4 OTHarmonizer workflow

#### 2.4.1 OT algorithm

The core idea of the OT algorithm is to define and solve a transport plan between two distributions, identifying the optimal way to transfer mass from one distribution to another while minimizing transportation costs. In this study, the source and target distributions are two batches of data, where each cell’s gene expression profile is treated as a data point with assigned weights to ensure total mass conservation.

Next, a cost matrix is constructed, where each element c(i, j) represents the cost of moving a point *i* from the source distribution to a point j in the target distribution, typically calculated based on the distance between the points (Euclidean distance is used here).

After determining the cost matrix, a transport matrix is defined to indicate how the mass from the source distribution is allocated to the target distribution. The goal of optimizing the transport matrix is to minimize the total transportation cost while satisfying the mass conservation constraint. To accelerate computation, OT often uses entropy regularization, which adds a small entropy term, allowing the problem to be efficiently solved using the Sinkhorn–Knopp algorithm, thus reducing computational complexity and making it suitable for large-scale data. Once optimized, the non-zero elements in the transport matrix represent the OT plan, indicating the actual transport paths and intensities from the source distribution to the target distribution.

In the application of OT, there are two main approaches: total transport and partial transport. Total transport maps all cells from the source batch to the target batch, while partial transport allows for only a subset of cells to be mapped. Performing a complete transport between two research batches may lead to over-matching, where new cell types in one batch are forcefully matched to unrelated cell types in the other batch. Partial transport provides a solution to this issue by allowing only a subset of cells to be mapped between batches, ensuring that alignment is restricted to biologically relevant matches. This approach helps prevent over-matching and ensures that unique or new cell types in one batch are not forcefully aligned to unrelated cell types in the other batch. In OTHarmonizer, we opted for partial transport because some studies may include cell types that do not exist in other studies. Forcing these cells to be mapped to annotations in another study is biologically unreasonable. By choosing partial transport, we provide the algorithm with the flexibility to identify and accommodate new cell types.

#### 2.4.2 Dataset ordering strategy

To obtain an optimized coarse-to-fine integration order, OTHarmonizer includes an optional batch-ordering strategy. Specifically, we first perform Leiden clustering at a fixed resolution on the fully integrated latent space, generating a global set of clusters shared across all batches. After clustering, we subset the clustered cells batch by batch, and for each batch B compute:

$N_{\mathrm{cluster}}(B)$ : number of Leiden clusters present in that batch;

$N_{\mathrm{label}}(B)$ : number of annotated cell types in that batch.

We then define the granularity ratio:


$$\mathrm{Granularity}\;\mathrm{Ratio}(B)=\frac{N_{\mathrm{cluster}}(B)}{N_{\mathrm{label}}(B)}$$

A higher ratio indicates coarser annotations (few labels covering many clusters), while a lower ratio indicates finer annotations. After computing this ratio for all batches, we sort them from high to low granularity (coarse to fine) and use this order during incremental hierarchy construction. This ensures that coarse batches establish the backbone first, and fine batches refine the hierarchy later, yielding a more stable and biologically coherent structure.

#### 2.4.3 Calculating transmission ratio matrix

In OTHarmonizer, batch annotation relationships are determined by pairwise comparisons of batches requiring harmonization. For any two batches of data, a partial transport approach is employed to compute the similarity matrix. The flexibility of partial transport is particularly suitable for cases where only a subset of cells between the source and target batches has matching annotations. To calculate the transport amount, the transport ratio is defined as follows:


$$\text{Transport}\;\text{Ratio}=0.4\times \frac{\min(|B_{1}|,|B_{2}|)}{\max(|B_{1}|,|B_{2}|)}$$

Here, $\left|B_{1}\right|$ and $\left|B_{2}\right|$ represent the number of cells in the source batch $B_{1}$ and the target batch $B_{2}$, respectively. This ratio limits the total amount of transport, helping to avoid over-matching by ensuring that unrelated cell types are not overly aligned with each other. This approach yields a cell transport plan matrix T from the source batch to the target batch.

To calculate the similarity between cell annotations, we normalize the transport plan for each source annotation group a (from $B_{1}$) and target annotation group b (from $B_{2}$). Let T(a, b) represent the number of cells transferred from annotation group a in $B_{1}$ to annotation group b in $B_{2}$. The similarity is then calculated as:


$$\text{Similarity}=\frac{T(a,\;b)}{\sum_{b^{\prime }}\;T(a,b^{\prime })}$$

where b' represents all target annotation groups in $B_{2}$. If a source annotation group a has no transport values, we set the similarity for that annotation group to zero:


$$\text{Similarity}=0,\;\text{if}\sum_{b^{'}}\;T(a,b^{\prime })=0$$

Using these formulas, we obtain the transmission ratio matrix S between the source and target batches, as well as a measure of similarity between cell annotation groups. This approach enables flexible alignment of cell annotations while avoiding over-matching.

#### 2.4.4 Deriving harmonization relationships

For the two batches being compared, each batch is alternately treated as the source batch to calculate the transmission ratio matrices, resulting in $S_{1}$ (from $B_{1}$ to $B_{2}$) and $S_{2}$ (from $B_{2}$ to $B_{1}$). In these matrices, each element $S_{1}(i,\;j)$ or $S_{2}(i,\;j)$quantifies the similarity between the annotation group in row i and the annotation group in column j based on gene expression. A higher S(i, j) value indicates a closer resemblance between the corresponding annotation groups. Using these matrices, the harmonization relationships between annotation groups across the two batches are determined according to the following rules:

1. *Parent–children relations (annotations with hierarchical relationships):* For a potential parent annotation group $c_{1}$ in $B_{1}$ and child annotations $c_{2},c_{3}\cdots c_{n}$ in $B_{2}$, if $S_{1}(c_{1},c_{2})>0$ indicating that $c_{1}$ contributes to $c_{2}$ in $B_{2}$, and $S_{2}(c_{2},c_{1})>0.65,\;$confirming a strong mapping from $c_{2}$ back to $c_{1}\;$(so as $c_{3}\cdots c_{n}$), then $c_{2},c_{3}\cdots c_{n}$ are considered as the subtypes of $c_{1}$.
2. *Equal relation (annotations with the same resolution and representing the same cell type)*: Equal annotations are determined by identifying annotation groups in the two batches that exhibit high mutual similarity in both transmission ratio matrices. Specifically, for an annotation group $c_{1}$ in $B_{1}$ and $c_{2}$ in $B_{2}$, if $S_{1}(c_{1},c_{2})>0.65$ and $S_{2}(c_{2},c_{1})>0.65$, indicating a strong similarity between $c_{1}$ and $c_{2}$, then $c_{1}$ and $c_{2}$ are considered as equal annotations. Additionally, $c_{1}$ and $c_{2}$ must not already be classified under Parent–child relations.
3. New types (annotations unique to one batch): New types are identified as annotation groups that are present in one batch but have no significant correspondence in the other batch. Specifically, an annotation group $c_{1}\in B_{1}$ is classified as a new type, if $\forall c_{2}\in B_{2},\;S_{1}(c_{1},c_{2})\leq 0.65\;\mathrm{and}\;S_{2}(c_{2},c_{1})\leq 0.65$. Similarly, an annotation group $c_{2}\in B_{2}$ is classified as a new type, if $\forall c_{1}\in B_{1},\;S_{2}(c_{2},c_{1})\leq 0.65\;\mathrm{and}\;S_{1}(c_{1},c_{2})\leq 0.65$.

#### 2.4.5 Building hierarchical trees

For the batches requiring harmonization, $B_{1},B_{2},\ldots ,B_{n}$, the process begins by calculating the harmonization relationships between the annotation groups in the first two batches, $B_{1}$ and $B_{2}$. Based on these relationships, the hierarchical tree is initialized following specific rules. First, the tree starts with a root node representing the most general category or label. Annotations classified as equal are merged into a single node, which is connected to the root node. Parent–children relationships identified between $B_{1}$ and $B_{2}$ are incorporated into the tree structure by linking parent nodes to their respective child nodes. Additionally, annotations classified as new types in either $B_{1}$ or $B_{2}$ are added as separate branches directly under the root node. This initialization lays the foundation for integrating additional batches into the hierarchical structure.

For the subsequent batch $B_{3}$, we perform harmonization with the corresponding nodes in the hierarchical tree and update the tree based on the obtained harmonization relations. This process follows a bottom-up comparison approach:

1. Select the child nodes directly connected to the root node, defined as Nodes={n|Parent(n)=Root}, and harmonize their corresponding cell groups with the annotation groups in $B_{3}$. At this step, we identify whether $B_{3}$ contains any new cell types, as the top-level nodes encompass all cell types in the hierarchical tree. Any new cell type annotations are directly added as child nodes of the root.
2. After removing the new cell types from $B_{3}$’s annotation groups, compare the remaining annotations with the cell groups corresponding to all leaf nodes in the tree, defined as Nodes={n∣Children(n)=∅}. The comparison follows these rules:
  - If an annotation $c_{3}$ in $B_{3}$ is equivalent to a leaf node, merge $c_{3}$ into that leaf node.
  - If $c_{3}$ is a subtype of a leaf node, add it as a child of that leaf node.
  - Define$\;C=\{c_{3\_\mathrm{new}}|c_{3\_\mathrm{new}}\;\text{is}\;a\;\text{new}\;\text{cell}\;\text{type}\}\cup \{c_{3\_\mathrm{pa}}|c_{3\_\mathrm{pa}}\;\text{is}\;a\;\text{parent}\;\text{node}\}$, and retain C and Nodes for the next step.
3. Compare C with the next higher-level nodes in the tree, defined as:
  Nodes={Parent(n)∣n∈Nodes, Parent(n)≠Root}∪{n∣Parent(n)=Root}

The comparison follows these rules:

- If $c_{3\_\mathrm{pa}}\in C$ is equivalent to a node in the current layer, merge them.
- If $c_{3\_\mathrm{pa}}\in C$ is a child of a node in the current layer, treat it as an intermediate node between the compared node and its child.
- If $c_{3\_\mathrm{new}}\in C$ is equivalent to or a child of a node in the current layer, add it directly as a new child of the compared node.
- Update $C=\{c_{3\_\mathrm{new}}|c_{3\_\mathrm{new}}\text{is}\;a\;\text{new}\;\text{cell}\;\text{type}\}\cup \{c_{3\_\mathrm{pa}}|c_{3\_\mathrm{pa}}\;\text{is}\;a\;\text{parent}\;\text{node}\}$, retain C and Nodes, and repeat step 3.

For subsequent batches, steps 1 to 3 are repeated iteratively, progressively harmonizing the annotations from each batch with the hierarchical tree. This process systematically integrates the annotations from all batches into the tree, achieving a comprehensive harmonization of all batch annotations.

### 2.5 Experiment setup

All experiments were conducted on a workstation equipped with dual Intel Xeon Gold 5320 CPUs (104 cores) and an NVIDIA A100 GPU (80 GB), with the system using CUDA 10.1. For OTHarmonizer, we used a subsampling size of 500 cells per annotation during OT-based matching, while other settings followed the standard harmonization pipeline. In all main OTHarmonizer experiments, the automatic dataset-ordering module was enabled, and datasets were ordered by the proposed granularity-based strategy rather than manually specified. Alternative orders were used only in the dataset-order ablation experiment, where the automatic coarse-to-fine order was compared with fine-to-coarse and random orders. scHPL and treeArches were trained using the scArches framework with SVM classifiers, dynamic neighbor selection enabled, and default thresholds for matching and rejection. For treeArches, we also tested kNN and one-class SVM settings; however, kNN often produced fragmented hierarchies by placing annotations as independent nodes rather than identifying equivalent relationships. Representative examples are shown in Fig. S10, available as supplementary data at *Bioinformatics* online, and we therefore used the best-performing SVM setting in the main benchmark.

Latent-space integration was conducted using scVI with the following configuration: 2 layers, 80 training epochs, 128 hidden units, 10 latent dimensions, 1 encoder layer, dropout rate of 0.1, gene-level dispersion, ZINB likelihood, and a normal latent distribution. CellHint was run with its key settings unchanged (default distance metric, PCT pruning, and automatic dataset ordering), as recommended by the original implementation. Remaining hyperparameters for all methods followed the authors’ defaults unless otherwise specified.

## 3 Results

### 3.1 Benchmarking framework for cross-dataset annotation harmonization

To evaluate the performance of existing methods for cell-type annotation harmonization, we designed a benchmark consisting of 10 scenarios across scRNA-seq datasets (Fig. 1). This benchmark includes five simulated scenarios generated by Symsim (Zhang *et al.* 2019b), three real-world scenarios focusing on harmonizing subtypes of specific cell types, and two more complex real-world scenarios on all cell types within specific organs, which aligning with the scenario of cell atlas construction.

We generated datasets with different annotation granularities, cell-type coverages, and batch effects, organizing them into five simulated scenarios (Fig. 1b, Section 2): coarse granularity, fine granularity, different granularity, mixed granularity, and different composition. Each scenario was designed to evaluate distinct aspects of harmonization performance, with pre-designed cell-type hierarchies serving as ground truth for the simulated datasets.

In the coarse granularity and fine granularity scenarios, the cell-type annotation granularity remained consistent across datasets, using a two-layer cell hierarchy with 13 cell types and subtypes. The coarse granularity scenario annotated all datasets at the upper layer, providing a simpler case to evaluate tools’ ability to align equivalence relationships while mitigating batch effects. In contrast, the fine granularity scenario annotated all datasets at the leaf nodes, challenging tools to capture more complex and detailed relationships.

The different granularity, mixed granularity, and different composition scenarios introduced more complex three-layer hierarchies to evaluate the ability to handle hierarchical relationships. In the different granularity scenario, we generated three datasets, each annotated to a different layer of the hierarchy. The mixed granularity scenario introduced a harder challenge by annotating each dataset with varying granularities across cell types, simulating the diverse research focuses across studies. For example, one dataset might provide fine-grained subtypes of immune cells but use a broader annotation for endothelial cells, while another might subdivide endothelial cells based on their location and function, but merge all subtypes of T cells, B cells, and natural killer cells into a general “Lymphocytes” label. The different composition scenario varied cell-type coverage across datasets, with each dataset containing a subset of the total cell types, testing the methods’ ability to identify novel cell types and incorporate them into the hierarchy.

The real-world scenarios involved a larger number of cell types and more complex relationships (Fig. 1c). We collected data from the constructed cell atlases of human lung and human heart, where original annotations were mapped onto pre-constructed hierarchies by experts. For the human lung data, we merged four datasets from HLCA to create a whole-lung scenario (Sikkema *et al.* 2023), and constructed three additional, relatively simpler scenarios focusing on subtypes of lymphocytes, endothelial cells, and epithelial cells. We also extracted three datasets of human heart data from hECA to form another atlas-level annotation harmonization scenario (Chen *et al.* 2022). In all five real-world scenarios, we used manually curated annotations as the ground truth and compared them with the hierarchies constructed by the automated tools.

We tested three existing methods—scHPL, treeArches, and CellHint—on these 10 scenarios and standardized their outputs into cell-type hierarchies (Fig. 1d). We designed three metrics to assess the results (Fig. 1e, Section 2): AH-F1, TEDS, and PCBS. AH-F1 is a metric directly constructed based on the macro-F1 score of the pairwise annotation relationship accuracy, designed to evaluate the correctness of the identified relationships between original annotations. TEDS measures overall similarity between constructed hierarchies and ground truths, while PCBS focuses on local structural similarity within the hierarchies and most sensitive to conditions with missing cell types. Additionally, we accessed the time consumption and scalability of each method in the real-world scenarios.

### 3.2 Overview of OTHarmonizer design

To address the challenge of cross-dataset annotation harmonization, we developed OTHarmonizer, a fast, accurate, and comprehensive tool leveraging OT. The workflow of OTHarmonizer consists of three-key steps: batch correction, annotation alignment, and hierarchy construction.

**Figure 2:**
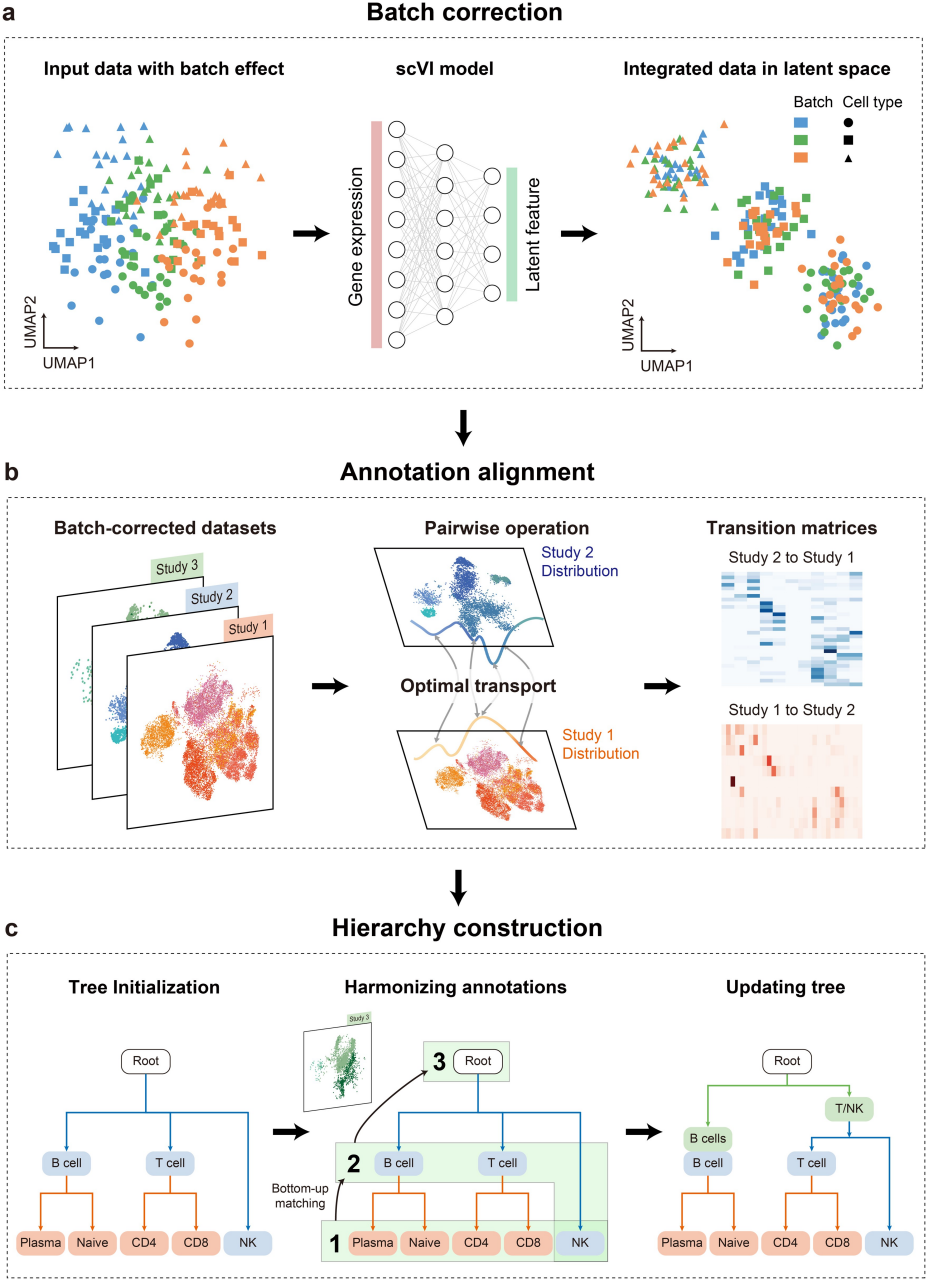
Workflow of OTHarmonizer. (a) Utilizing scVI to correct batch effects and extract integrated latent features. (b) Optionally ordering datasets by annotation granularity, followed by identifying annotation relationships between datasets using Optimal Transport, which computes pairwise correspondences and generates transition matrices. (c) A hierarchy is initialized based on the annotation relationships from the first two batches. For subsequent batches, cell annotations are matched to the existing hierarchy using a bottom-up approach, progressively updating the hierarchy with relationships across all prior batches.

In the first step, OTHarmonizer mitigates batch effects across studies (Fig. 2a). Recognizing the critical role of batch correction, we utilize scVI to extract a unified and robust latent space (Section 2). This ensures that annotation alignment prioritizes biological gene expression similarities over technical noise. The second step aligns annotations between datasets. Before performing pairwise OT matching, OTHarmonizer can optionally order datasets by annotation granularity (Fig. 2b), arranging them from coarse to fine resolution to promote more stable and interpretable alignment. After, OTHarmonizer employs OT to compute pairwise correspondences between latent space distributions (Fig. 2b). By minimizing transition costs, OTHarmonizer effectively aligns cells and reveals annotation relationships using a matching strategy (Section 2). In the final step, OTHarmonizer constructs an annotation hierarchy based on these relationships. The hierarchy is initialized with annotations from the first two datasets. For subsequent datasets, new annotations are iteratively matched to existing nodes, starting at leaf nodes and progressing upward. Each matching step applies the OT-based method described earlier, ensuring that the hierarchy evolves to accurately reflect cell type relationships (Fig. 2c). This design naturally supports incremental integration of additional datasets, allowing the hierarchy to expand seamlessly as new data become available.

### 3.3 Harmonization performance in the simulated scenarios

**Figure 3:**
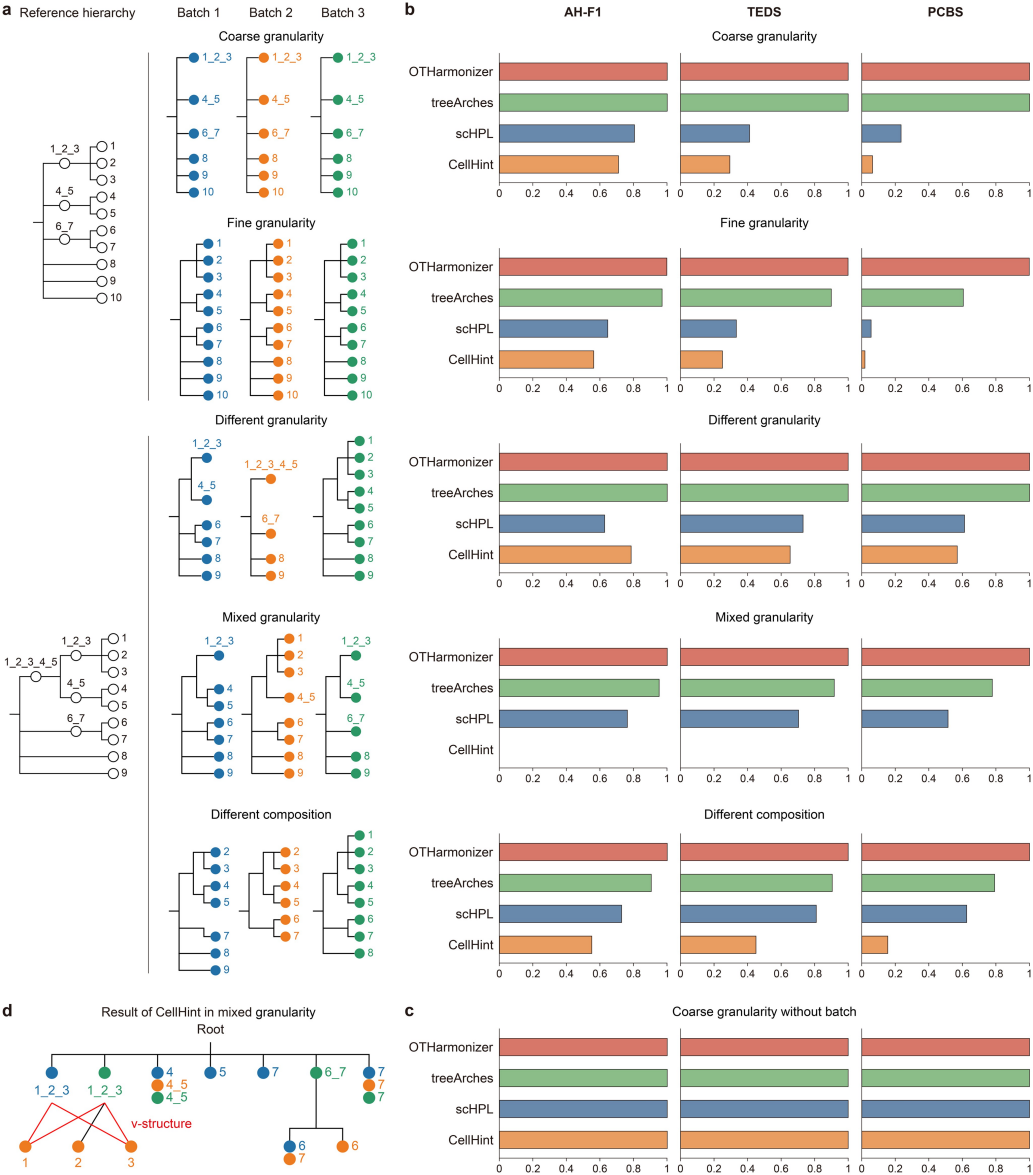
Harmonization performance of the automatic tools in five simulated scenarios. (a) The reference hierarchies and the hierarchies in three generated batches across five simulated scenarios. (b) The bar charts of AH-F1, TEDS, PCBS scores across the five simulated scenarios in our benchmark. (c) The performance comparison of the four methods under coarse granularity without batch effect. (d) The hierarchy produced by CellHint in the mixed granularity scenario, with the v-structure highlighted with red lines.

During annotation harmonization, tools face multiple challenges simultaneously, such as inconsistencies in annotation granularity, discrepancies in cell type definitions, variations in subtype annotation standards, and batch effects. Our benchmark evaluates each tool’s performance in addressing these challenges, focusing on one specific issue in each simulated scenario (Fig. 3a; Fig. S1, available as supplementary data at *Bioinformatics* online).

The benchmark results were shown in Fig. 3b. In the coarse granularity scenario, treeArches outperformed the other two tools, accurately aligning equivalence relationships between coarse annotations despite batch effects. Since the key difference between treeArches and its predecessor, scHPL, lies in the additional data integration with scVI (Xu *et al.* 2021), we conducted an ablation experiment to investigate the source of its superior performance. We re-generated a set of datasets with coarse annotations but without batch effects (Section 2). When evaluated under this condition, all methods achieved high scores (Fig. 3c), demonstrating their ability to align reliably with minimal batch effects. This result highlights the importance of batch effect correction before alignment. Although the PCT algorithm in CellHint is reported to be robust against batch effects, it could not withstand strong batch effects (Xu *et al.* 2023).

In the five simulated scenarios, OTHarmonizer consistently achieved the highest scores (Fig. 3b). In the fine granularity scenario, all methods except OTHarmonizer exhibited a performance drop (Fig. 3b). The finer annotation granularity led to higher similarity among gene expression profiles of subtypes, making it more challenging for the classifiers in these methods to distinguish them. This resulted in misclassified relationships and relatively low PCBS scores.

Across the different granularity, mixed granularity, and different composition scenarios, OTHarmonizer and treeArches outperformed scHPL and CellHint, benefiting from its robust batch effect correction. Both treeArches and scHPL showed similar performance in the mixed granularity and different composition scenarios, indicating their capability to identify novel cell types and prevent misalignments. In contrast, CellHint struggled in the mixed granularity scenario, failing to construct a proper cell hierarchy. It assigned multiple parent cell type nodes to subtype nodes, forming a v-structure that converted the aligned relationships into a directed acyclic graph rather than a tree structure (Fig. 3d). This issue arose from CellHint’s tendency to directly match new study’s annotations to the leaf nodes in the hierarchy.

### 3.4 Harmonization performance in the cell-type-specific real-world scenarios

To evaluate whether annotation harmonization tools meet the requirements for real-world applications, we assessed their performance in real-world scenarios. Unlike simulated datasets, real scRNA-seq data exhibits greater cell-type diversity, more significant heterogeneity, more complex relationships, and more pronounced batch effects. We focused on three representative cell populations from HLCA (Sikkema *et al.* 2023): endothelial cells, epithelial cells, and immune cells. These cell types are important due to their roles in lung disease and immune response—endothelial cells contribute to vascular structure and function, epithelial cells form the lung tissue barrier, and immune cells play a vital role in immune defense and inflammation (Brune *et al.* 2015, Rankin and Artis 2018, Augustin and Koh 2024). Accurately distinguishing subpopulations within these cell types and preserving their hierarchical relationships are critical for constructing a comprehensive lung cell atlas and understanding disease mechanisms.

**Figure 4:**
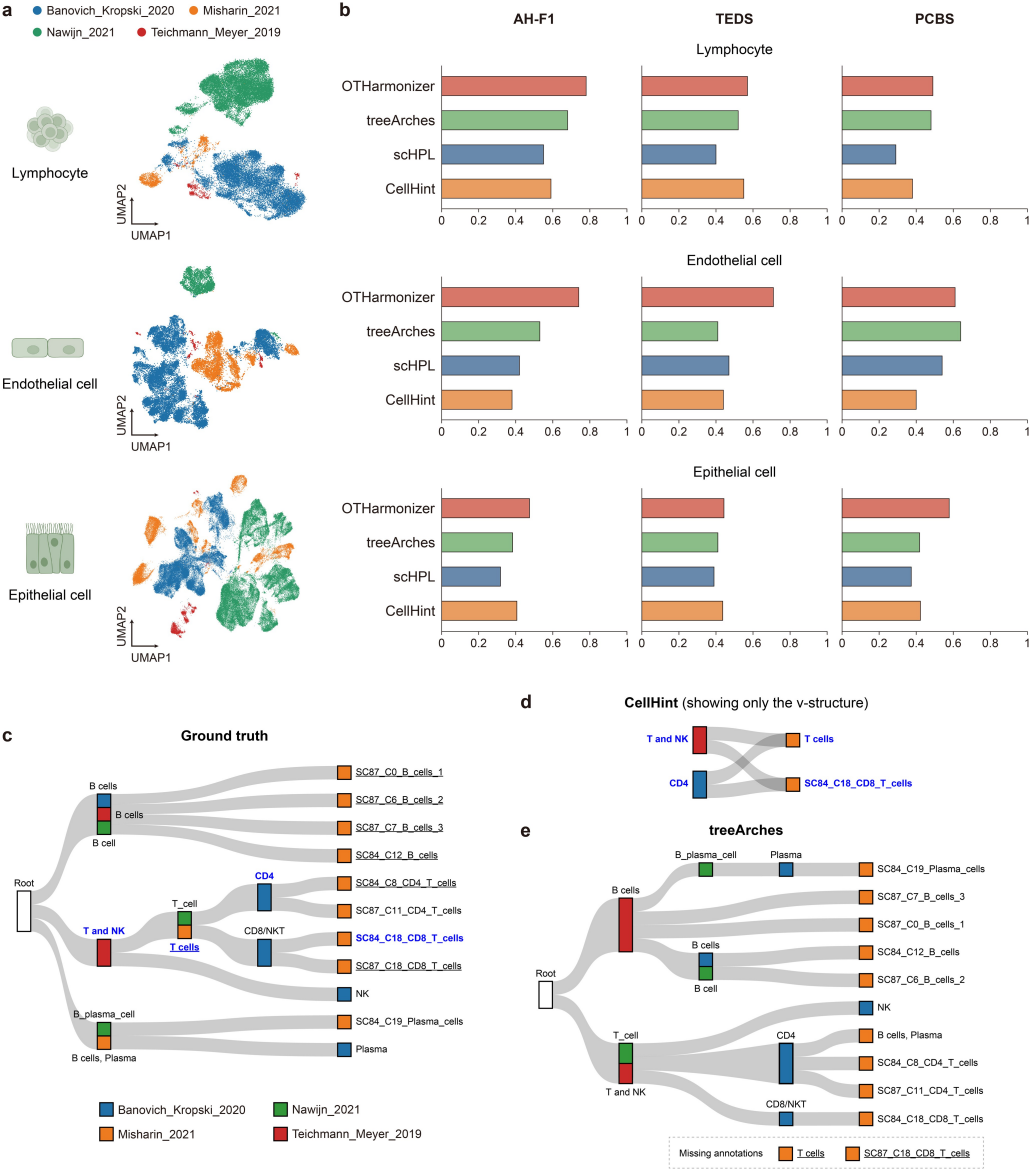
Harmonization performance of the automatic tools in three cell-specific real scenarios. (a) UMAP visualizations of real scenarios on lymphocytes, endothelial cells and epithelial cells in human lung, colored by batches. (b) The AH-F1, TEDS, PCBS scores across the automatic tools in the three scenarios. (c) The ground truth hierarchy of the lymphocyte scenario. Cell types showing the v-structure in (d) are colored with blue, and cell types missed in (e) are highlighted with underline. (d) The hierarchy produced by CellHint in the lymphocyte scenario, showing only the v-structure. (e) The hierarchy produced by treeArches in the lymphocyte scenario.

We evaluated the performance of four harmonization tools—OTHarmonizer, scHPL, TreeArches, and CellHint—on datasets from four batches in the HLCA: Banovich_Kropski_2020, Teichmann_Meyer_2019, Nawijn_2021, and Misharin_2021 (Fig. 4a). These datasets contain samples from diverse individuals, leading to strong batch effects and varying levels of annotation resolution (Fig. S2, available as supplementary data at *Bioinformatics* online). For example, Teichmann_Meyer_2019 provides only broad cell-type annotations, while Misharin_2021 includes more granular subtype classifications. These discrepancies in annotation granularity pose challenges for harmonization.

Our results revealed notable differences among the four methods across the three cell-type-specific scenarios (Fig. 4b). Overall, OTHarmonizer achieved the best performance, while treeArches generally performed better than scHPL and CellHint among the existing tools. In terms of AH-F1, treeArches showed comparable or better performance than scHPL and CellHint, suggesting that scVI-based integration helped reduce the impact of batch effects. In contrast, scHPL generally underperformed, indicating its sensitivity to batch variation. However, the existing methods still showed limited preservation of global and local hierarchy structures, with TEDS and PCBS scores mostly below 0.6. Compared with these methods, OTHarmonizer showed a clear advantage in the endothelial scenario and achieved comparable or better performance in the lymphocyte and epithelial scenarios, especially in AH-F1, suggesting that it more accurately captured annotation-level relationships across datasets while maintaining competitive hierarchical structure recovery.

We took the lymphocyte scenario as an example to further investigate the harmonization results. CellHint, in particular, produced a “v-structure” in the lymphocyte scenario (Fig. 4c and d). In this structure, the annotations SC84_C18_CD8_T_cells and T cells from Misharin_2021 shared two common parent nodes: T and NK from Teichmann_Meyer_2019 and CD4 from Banovich_Kropski_2020. This structure lacked full equivalency or hierarchical relationships among these nodes, leading to the loss of higher-level information and preventing the formation of a complete hierarchical tree. Due to this structure, the harmonization result was no longer a tree. To facilitate comparison, we combined the parent nodes in the v-structure into equivalent nodes and reconstructed a hierarchical tree for comparison with the ground truth. Notably, neither scHPL nor treeArches exhibited this issue. Besides, though treeArches demonstrated stronger harmonization of immune cells, it excluded two annotations from the Misharin_2021 dataset during harmonization, resulting in an incomplete hierarchical tree and affecting the alignment’s overall consistency and completeness (Fig. 4e).

Overall, OTHarmonizer improved the preservation of local hierarchical structures in cell-type-specific real-world scenarios. Nevertheless, remaining errors indicate that maintaining both global topology and local branch relationships remains challenging when annotation resolution varies across real datasets.

### 3.5 Harmonization performance in the atlas-level real-world scenarios

**Figure 5:**
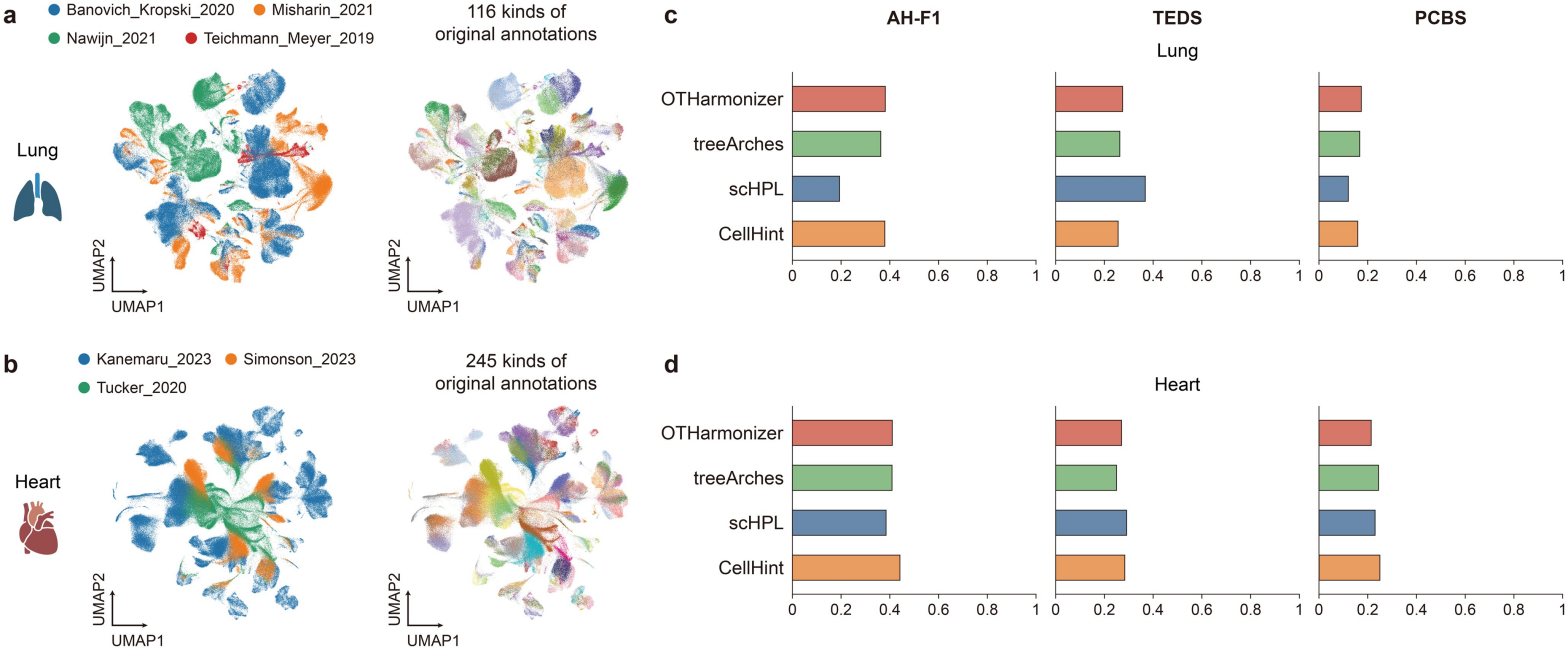
Harmonization performance of the automatic tools in the two organ-level real scenarios. (a) UMAP visualizations of the human lung datasets, colored by batches and original annotations. (b) The AH-F1, TEDS, PCBS scores across the automatic tools in the real lung scenario. (c) UMAP visualizations of the human heart datasets, colored by batches and original annotations. (d) The AH-F1, TEDS, PCBS scores across the automatic tools in the real heart scenario.

Annotation harmonization tools face significant challenges when applied to atlas-level data during cell atlas construction. These scenarios involve highly complex biological contexts, a large number of original annotations, and substantial increases in the number of cells. To test these conditions, we included two atlas-level real-world resources, HLCA (Sikkema *et al.* 2023) and hECA (Chen *et al.* 2022). From these resources, we selected seven datasets for our benchmark. The HLCA collection contributes Banovich_Kropski_2020 (Habermann *et al.* 2020), Misharin_2021 (Bharat *et al.* 2020), Teichmann_Meyer_2019 (Vieira Braga *et al.* 2019), and Nawijn_2021 (Vieira Braga *et al.* 2019). The hECA collection contributes Kanemaru_2023 (Kanemaru *et al.* 2023), Tucker_2020 (Tucker *et al.* 2020), and Simonson_2023 (Simonson *et al.* 2023) (Fig. 5a and b).

All evaluated methods, including OTHarmonizer, showed limited performance in the atlas-level scenarios (Fig. 5c and d). The poor results can be attributed to the large number of annotation labels in atlas-level datasets, which pose significant challenges for the multi-class classifiers or similarity-predicting models used by current methods. These challenges lead to frequent misassignments, reflected in lower AH-F1 scores. Additionally, the increased complexity of hierarchical structures amplifies the impact of misassignments. Even minor errors can lead to substantial differences in the overall tree structure, causing very low TEDS scores. As the scale of scRNA-seq datasets grows, the impact of batch effects also intensifies, further complicating annotation alignment and further degrading local matching performance.

While scHPL showed relatively better performance in terms of TEDS, further analysis revealed that its higher score was primarily due to a conservative strategy of discarding a large number of annotations. This approach reduces the likelihood of structural errors in the hierarchy but compromises overall integration performance. This limitation is evident in the PCBS metric, where scHPL fails to generate a complete or robust hierarchical structure.

These results underscore the limitations of current tools. None are fully capable of supporting an end-to-end automated process for annotations alignment, data integration, and cell-type hierarchy construction. Experts and manual curation on the tool outputs remain essential. These findings highlight the substantial challenges and unmet demands in this field, underscoring the need for further methodological innovations.

### 3.6 Enhancing harmonization accuracy and speed with OTHarmonizer

We evaluated OTHarmonizer across 10 benchmark scenarios. In the five simulated scenarios, OTHarmonizer consistently achieved the highest scores (Fig. 3b). In cell-type-specific real-world scenarios involving three human lung populations, OTHarmonizer outperformed existing tools (Fig. 4b). Notably, in lymphocyte-specific datasets, OTHarmonizer improved performance by over 0.2 across all metrics. For the other two cell-type-specific datasets with greater heterogeneity and more complex subtypes, OTHarmonizer achieved comparable or slightly better results in AH-F1 and TEDS metrics but showed significant gains in PCBS. This highlights OTHarmonizer’s ability to detect fine-grained subtypes and reconstruct local structures within hierarchies.

However, like existing tools, OTHarmonizer struggled with atlas-level datasets. For the lung atlas, OTHarmonizer performed best in AH-F1 and PCBS but lagged behind scHPL in TEDS (Fig. 5c). For the heart atlas (Fig. 5d), all tools showed similar performance, suggesting that residual batch effects and cellular heterogeneity continue to impede alignment, even with scVI integration.

To validate the biological correctness of the harmonization results, we invited researchers with extensive experience in cell type annotation to perform a blind review of the endothelial hierarchies, as illustrated in Fig. S3, available as supplementary data at *Bioinformatics* online. Without knowing which method produced each tree, the researchers compared the outputs of treeArches and OTHarmonizer and consistently judged the OTHarmonizer hierarchy to be more complete and biologically coherent. They noted that treeArches missed several key endothelial annotations, including Aerocyte/Artery, Bronchial_endothelial, and Bronchial/Venous, which led to major gaps in the reconstructed lineage structure. In contrast, OTHarmonizer successfully retained all annotations and produced a more faithful hierarchy with only minor topological inconsistencies. The assessments aligned closely with the quantitative evaluation: treeArches received a lower AH-F1 score (0.675), while OTHarmonizer achieved a higher score (0.775), confirming that the proposed metric reflects biological accuracy in practice. This improvement suggests that the partial OT step helps OTHarmonizer preserve annotations with partial or weak correspondence across datasets, rather than leaving them unmatched during hierarchy reconstruction.

We further examined the impact of dataset addition order in real scenarios and evaluated the effectiveness of our coarse-grained ordering strategy. We compared three integration sequences: coarse-to-fine, which begins with datasets containing broad, high-level cell categories (the strategy used by OTHarmonizer); fine-to-coarse, which begins with datasets containing highly detailed annotations; and random. We found that the coarse-to-fine order consistently produced the most stable and accurate hierarchies across both lymphocyte and endothelial evaluations (Fig. S4, available as supplementary data at *Bioinformatics* online). Establishing coarse cell categories first provides a robust structural scaffold, enabling finer subtypes to be aligned more reliably in later steps. In contrast, adding fine-grained datasets early often disrupts higher level relationships, leading to reduced PCBS and AH-F1 scores. These results demonstrate the importance of using an appropriate dataset order for incremental harmonization and confirm that our automatic coarse-grained ordering algorithm offers a principled and effective solution.

We assessed how integration quality affects annotation harmonization by evaluating TEDS, PCBS, and AH-F1 across latent spaces with different levels of batch mixing (Section 2). As shown in Fig. S5a, available as supplementary data at *Bioinformatics* online, all three metrics increase with Batch Average silhouette width (ASW), indicating that better integration leads to more accurate hierarchical recovery. The corresponding hierarchy reconstructions in Fig. S5b, available as supplementary data at *Bioinformatics* online, further illustrate this trend: when Batch ASW is low, the tree contains extensive misplacements; at intermediate integration, the structure becomes more coherent; and at high ASW, the hierarchy closely matches the ground truth. These results show that harmonization accuracy is strongly dependent on integration quality, and effective batch correction is essential for reliably aligning fine-grained cell types.

**Figure 6:**
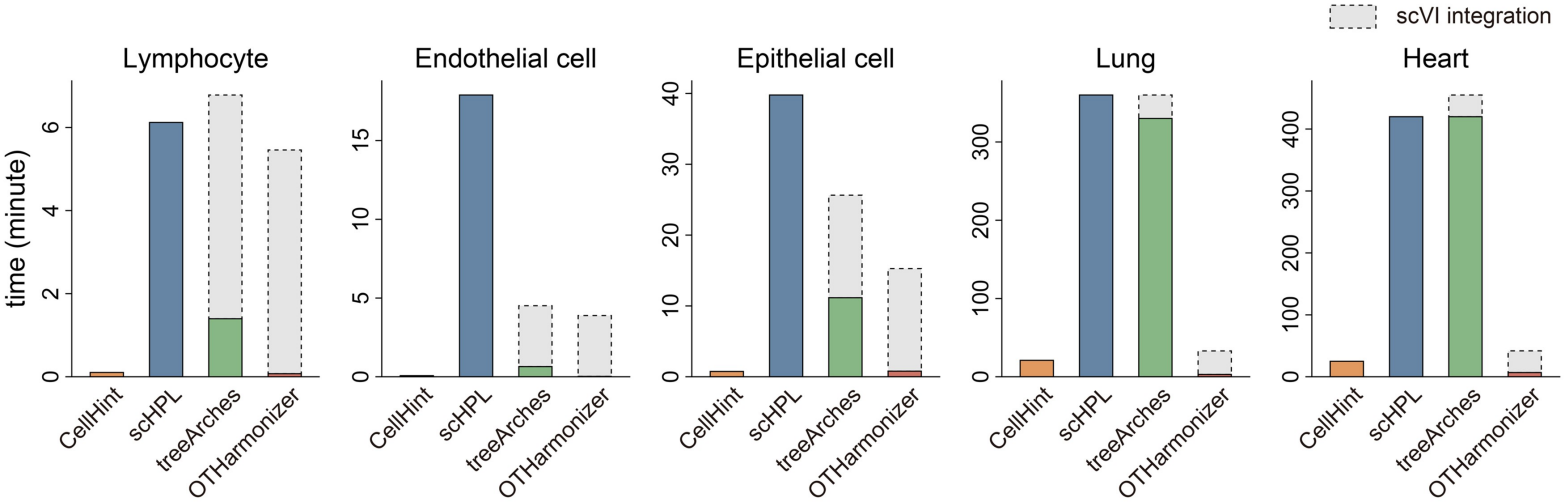
The computational efficiencies of harmonization tools in five real scenarios. For treeArches and OTHarmonizer, the dashed bars represent the time spent on data integration via scVI.

OTHarmonizer demonstrated significant advantages in computational efficiency (Fig. 6). Across the five real-world scenarios, OTHarmonizer processed large-scale datasets up to 100 times faster than scHPL and TreeArches. This efficiency stems from the use of fast numerical algorithms for OT, such as the Sinkhorn–Knopp algorithm and entropy-regularized methods (Bonneel *et al.* 2011, Cuturi 2013). These optimizations reduce the computational complexity of large-scale transport problems, enabling rapid annotation alignment and hierarchy construction.

We also examined another scenario in which datasets adopt mutually incompatible annotation criteria (Fig. S6a, available as supplementary data at *Bioinformatics* online). In this controlled simulation, the two batches shared the same underlying structure but were partitioned along orthogonal directions, creating label sets that partially overlap biologically yet cannot be aligned structurally. We found that existing methods exhibit clear limitations in this setting (Fig. S6b, available as supplementary data at *Bioinformatics* online): scHPL produces an overly shallow hierarchy, CellHint interprets weak overlaps as nested relationships, and OTHarmonizer, although better at capturing intersections, still cannot fully reconcile fundamentally inconsistent annotation schemes. These observations indicate that when annotation principles differ conceptually, purely data-driven harmonization reaches an intrinsic limit.

We further examined a non-nested mixed-granularity case commonly observed when annotations are derived from clustering at different resolutions (Fig. S8, available as supplementary data at *Bioinformatics* online). In this setting, different datasets may partition the same underlying populations as [1,2] and [3], [1] and [2,3], or [1], [2], and [3]. This structure is not strictly tree-consistent because [1,2] and [2,3] overlap at population 2, but neither is a subset of the other. Therefore, all tree-based methods can only provide an approximate hierarchy. Importantly, OTHarmonizer additionally reports bidirectional OT transport proportions, which reveal how shared mass is distributed across partially overlapping annotations and provide useful evidence for interpreting such non-nested relationships.

In summary, OTHarmonizer stands out as a leading tool for cross-dataset annotation harmonization, offering superior accuracy and computational efficiency. These capabilities make it an efficient tool for large-scale single-cell studies.

## 4 Discussion

Harmonization tools are essential for integrating single-cell datasets from diverse sources and constructing comprehensive cell atlases. By systematically aligning cell type annotations across datasets, they significantly reduce reliance on manual efforts, making the data analysis process faster and more scalable. These tools are also capable of uncovering hierarchical relationships between annotations, facilitating the construction of layered cell type reference trees and providing deeper insights into cellular organization. Moreover, these tools offer significant value in disease biology by identifying shared and distinct cell populations across studies (Xu *et al.* 2023). This capability allows them to reveal hierarchical relationships among cell subtypes involved in disease progression, including transitional states, thus contributing critical insights into the underlying mechanisms of disease.

Despite their progress, this benchmarking study suggested that all harmonization tools face notable limitations when addressing complex real-world data scenarios, particularly atlas-level datasets. Such datasets are characterized by high annotation complexity, large cell numbers, and substantial batch effects. While OTHarmonizer leverages scVI for batch effect correction and employs OT for efficient alignment of cell annotations, it performs suboptimally on large-scale datasets, exposing inherent shortcomings in current methods.

One major limitation is the tendency of most harmonization tools to focus on annotation matching between batches while neglecting hierarchical structures within individual batches. This oversight leads to the simplification of intra-batch cell subtypes and parent–child relationships into flat structures during integration, failing to accurately reflect biological hierarchies. For instance, in the HLCA dataset, the T cell annotations from Misharin_2021 include multiple levels, such as T cells, SC87_C11_CD4_T_cells, SC84_C8_CD4_T_cells, SC84_C18_CD8_T_cells, and SC87_C18_CD8_T_cells. The latter four are subtypes of the first annotation, but current integration methods often disregard these intra-batch hierarchical relationships, resulting in discrepancies between integration outputs and actual biological structures. This issue not only reduces the biological accuracy of the integration but also limits a comprehensive understanding of cell hierarchical relationships.

Additionally, existing harmonization tools exhibit high sensitivity to threshold selection and parameter settings, which undermines their flexibility when processing complex datasets. For example, in scenarios with strong batch effects, high annotation resolution, or highly complex cell types, fixed thresholds may significantly reduce annotation matching accuracy, increasing integration errors and risking the loss of critical biological information.

Another limitation of harmonization arises when datasets use fundamentally different annotation principles. Unlike differences in granularity, these discordant schemes reflect orthogonal concepts, such as functional states versus spatial locations, that do not form hierarchical inclusion. Although the labels may overlap biologically, they cannot be unified into a coherent hierarchy using data alone, revealing an intrinsic boundary of current harmonization approaches. Resolving such inconsistencies will likely require the incorporation of external biological knowledge or curated ontologies.

Future advancements in harmonization tools should address these challenges by prioritizing flexibility and adaptability. Developing algorithms with dynamic parameter adjustment mechanisms could improve generalization capabilities, enabling tools to accommodate varying dataset complexities and annotation resolutions. Moreover, integrating additional biological information, such as temporal dynamics and functional characteristics, will enhance the ability to capture functional states of cells, particularly for identifying subtle subtypes and transitional states.

The benchmark results highlighted the critical role of data integration in annotation harmonization. This dependency is especially important when the integration error is not merely insufficient alignment but incorrect alignment (Fig. S7, available as supplementary data at *Bioinformatics* online). OTHarmonizer performs OT-based matching in the integrated space, while TreeArches also relies on the neighborhood structure after integration. Therefore, if biologically unrelated populations are incorrectly mixed during upstream integration, downstream harmonization methods may infer artificial equivalence or hierarchical relationships from these false neighborhoods.

Recently, large-scale cellular language models have emerged with the capability to effectively integrate data (Yang *et al.* 2022, Cui *et al.* 2024, Hao *et al.* 2024), offering new opportunities for improved annotation harmonization. Furthermore, harmonization can serve as a standardized benchmark to evaluate the performance and effectiveness of foundation models.

## Supplementary Material

btag506_Supplementary_Data

## Data Availability

The simulated and real-world datasets in the benchmark are available on https://figshare.com/articles/dataset/OTHarmonizer/28243205. The source codes for the benchmark and OTHarmonizer are available online on GitHub at https://github.com/Duck-Boss/OTHarmonizer.
