# Supplementary material for: Cross-dataset annotation harmonization for cell-type hierarchy construction
Source: Bioinformatics. 2026 Jul 9;42(7):btag506. doi: 10.1093/bioinformatics/btag506 (PMC13401821; doi:10.1093/bioinformatics/btag506)
Supplement: btag506_Supplementary_Data [file btag506_supplementary_data.pdf]

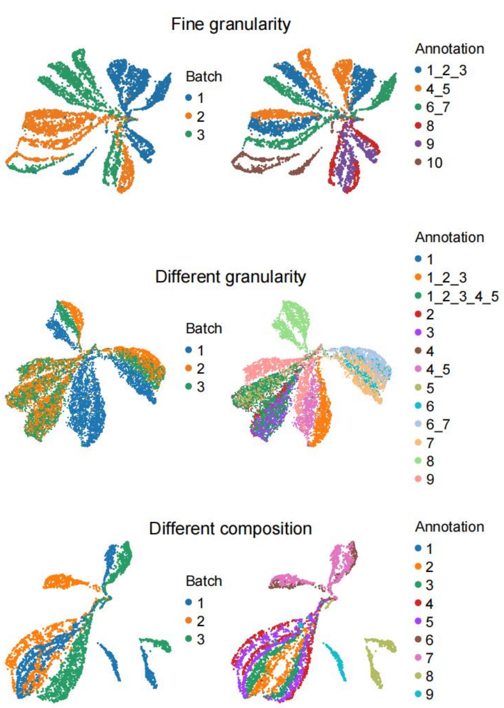

Fig. S1. The UMAP visualization of the generated data in the five simulated scenarios, colored by batch and annotations.

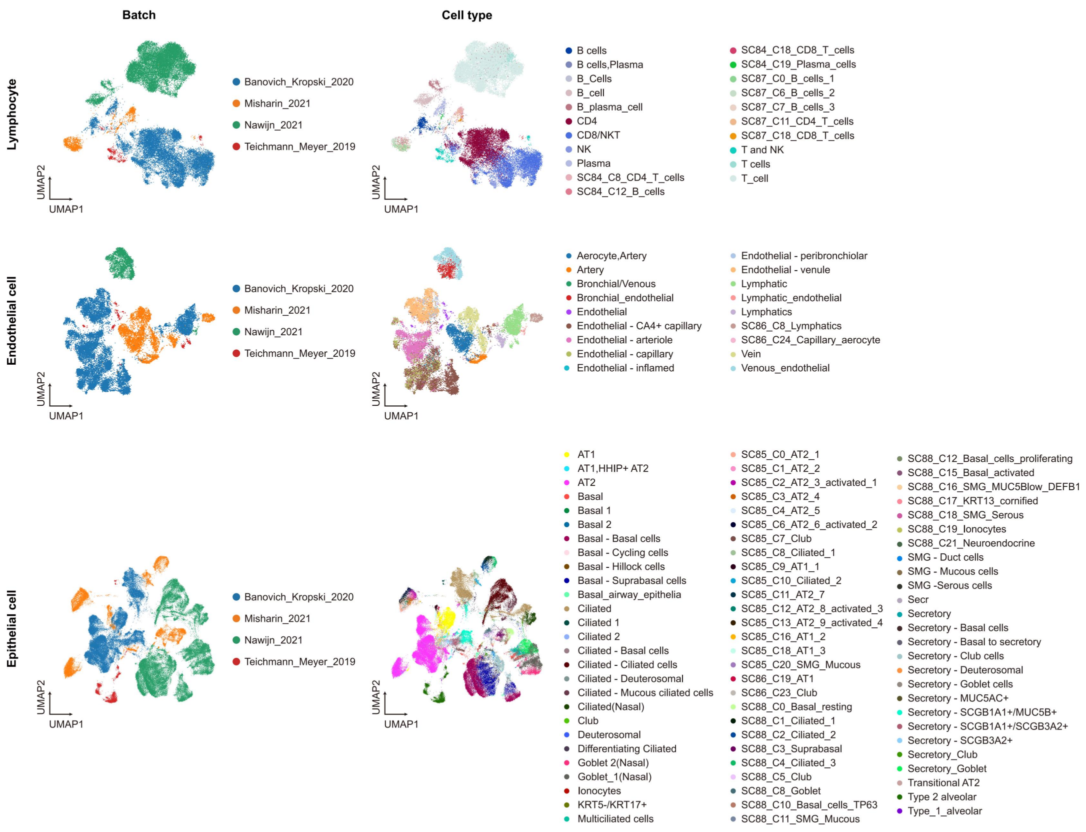

Fig. S2. The UMAP visualization of the data in three cell-type specific real scenarios, colored by batches (left) and original annotations (right).

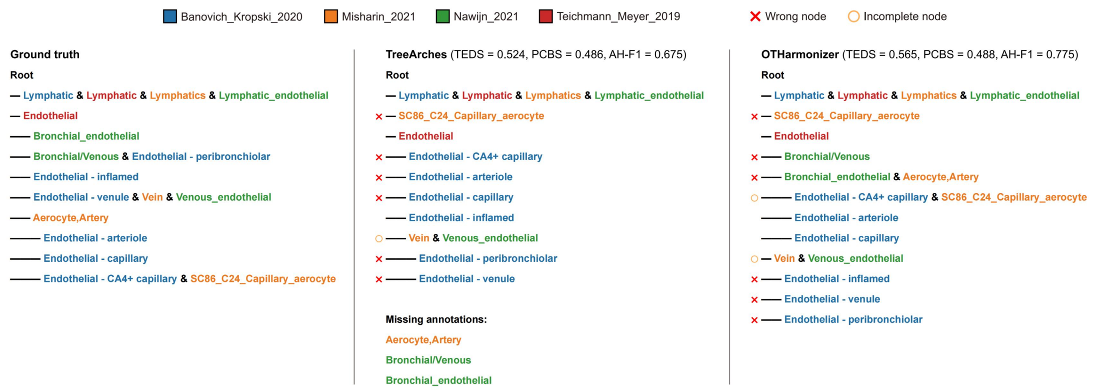

Fig. S3. Comparison of ground-truth, treeArches, and OTHarmonizer harmonized endothelial hierarchies, highlighting differences in completeness and structural consistency across datasets.

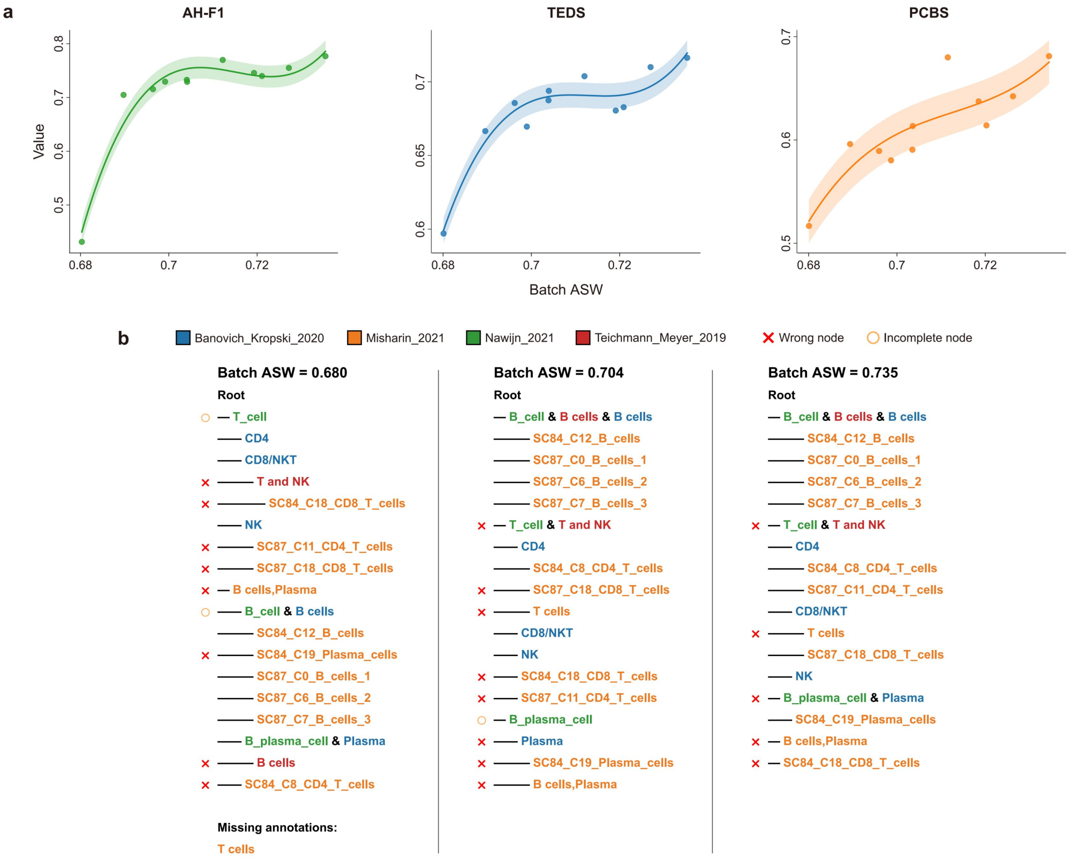

Fig. S4. Impact of integration quality on harmonization performance. (a) Relations between integration quality (Batch ASW) and harmonization metrics (TEDS, PCBS, AH-F1). Shaded regions indicate confidence intervals. (b) Example hierarchies obtained under different levels of batch integration.

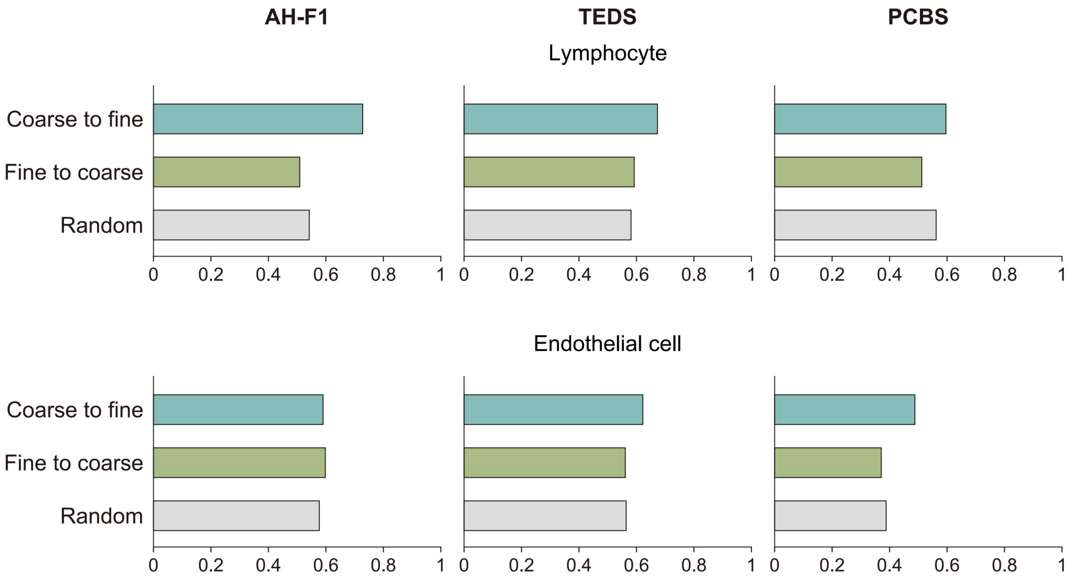

Fig. S5. Quantitative comparison of hierarchy construction performance across different dataset addition orders.

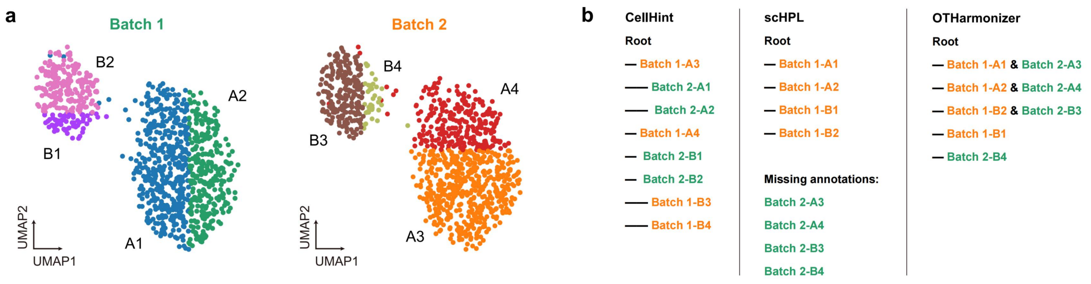

Fig. S6. Impact of orthogonal annotation schemes on automated hierarchy construction.

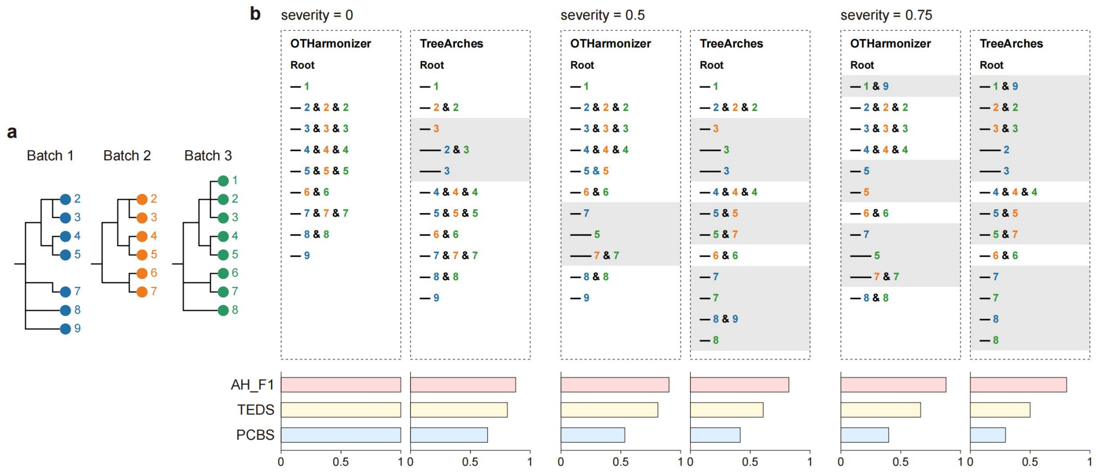

Fig. S7. Incorrect-integration stress test in the different-composition simulation.

(a) Ground-truth hierarchy of the different-composition simulation.

(b) Inferred hierarchies and evaluation scores of OTHarmonizer and TreeArches under increasing incorrect-integration severity. We simulated incorrect integration by progressively moving two unrelated populations, 9-batch 1 and 1-batch 2, toward a shared latent center in the scVI latent space:

$$z_i^{(\alpha)} = (1-\alpha)z_i + \alpha c + \epsilon_i, \qquad c = \frac{\mu_{9-\text{hatch }1} + \mu_{1-\text{batch }2}}{2}.$$

Here, α is the severity coefficient, z_i is the original latent embedding, c is the shared latent center, and ϵ_i is a small noise term. α=0 denotes the original latent space, while larger α values create stronger artificial mixing. Highlighted nodes indicate spurious relationships caused by incorrect integration.

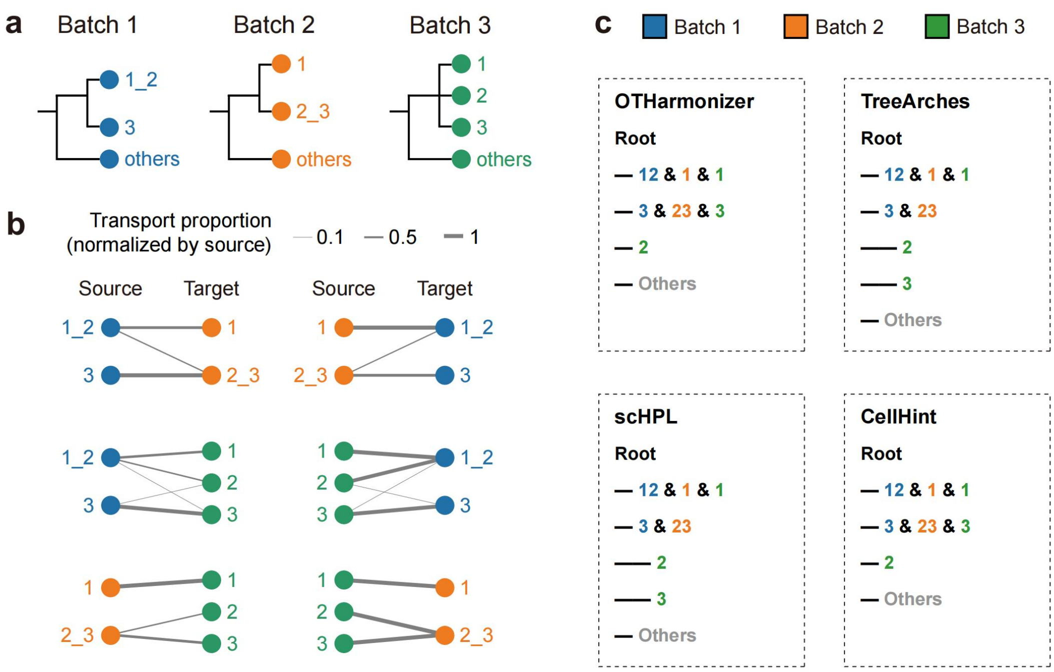

Fig. S8. Non-nested mixed granularity revealed by OT transport proportions.

(a) Schematic of the non-nested mixed-granularity simulation. The same underlying populations are annotated differently across batches: Batch 1 as [1,2] and [3], Batch 2 as [1] and [2,3], and Batch 3 as [1], [2], and [3]. This creates a non-tree structure because [1,2] and [2,3] overlap at population 2.

(b) Source-normalized OT transport proportions from OTHarmonizer. Edge widths indicate the fraction of transport mass between annotations, revealing partial correspondences across non-nested partitions.

(c) Hierarchies inferred by OTHarmonizer, TreeArches, scHPL, and CellHint. Since this case is not strictly tree-consistent, all methods can only provide approximate tree representations, while OT transport proportions provide additional evidence for interpreting partial-overlap relationships.

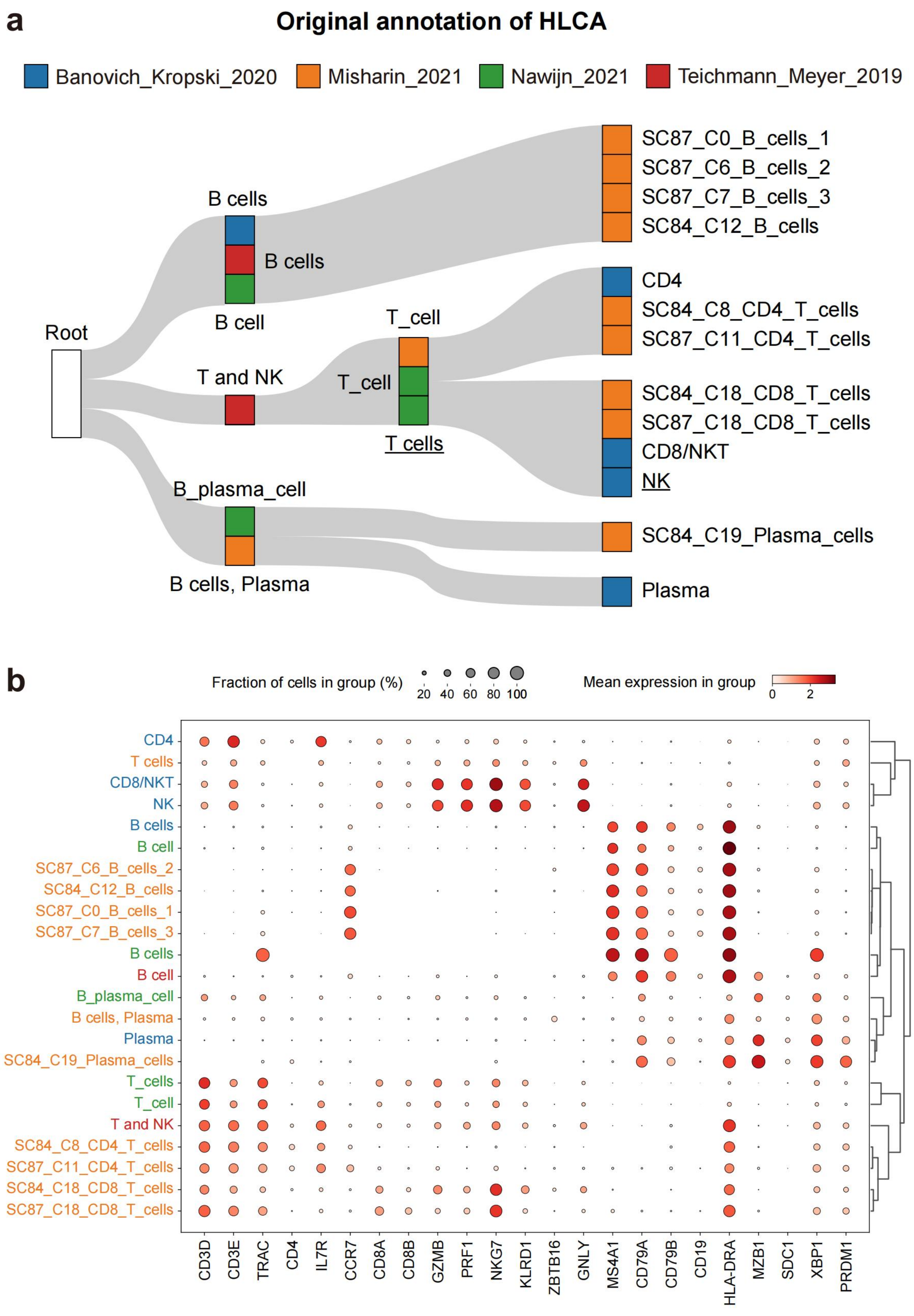

Fig. S9. Marker-supported minimal refinement of the HLCA lymphocyte hierarchy.

(a) Original HLCA lymphocyte hierarchy used as the starting point for ground-truth

construction. Different colors indicate the source datasets of the annotations.
(b) Dot plot of canonical marker genes curated from CellMarker 2.0 and related references. Marker expression supports minor refinements of the original hierarchy, including resolving duplicated T-cell labels and separating over-merged NK-related annotations from the CD8/NKT branch. These refinements were restricted to clear marker-supported inconsistencies, while the remaining HLCA hierarchy was kept unchanged.

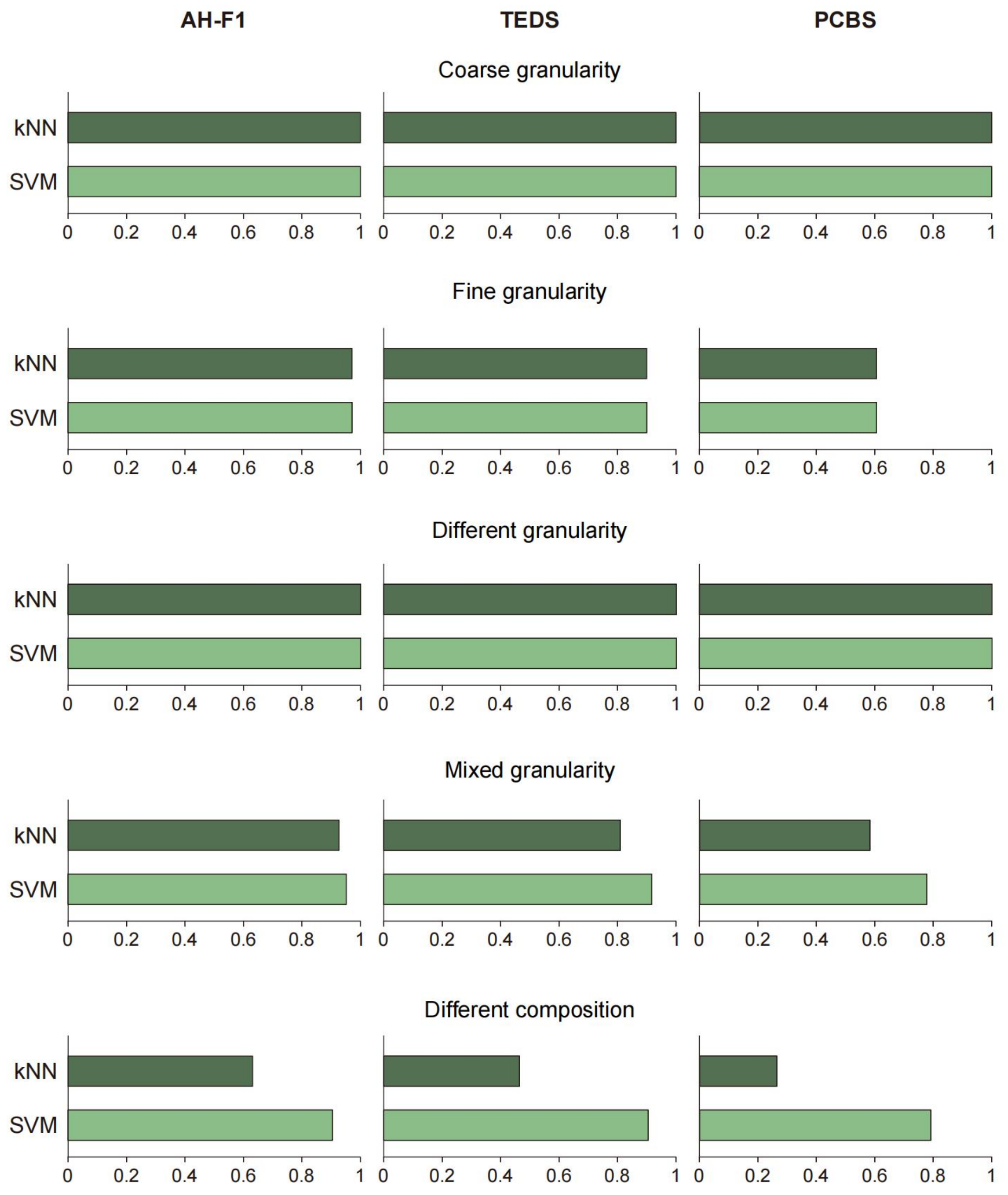

Fig. S10. Comparison of treeArches-kNN and treeArches-SVM in simulated scenarios.

AH-F1, TEDS, and PCBS scores of treeArches using kNN and SVM classifiers across five simulated benchmark scenarios. Although kNN is recommended in the original treeArches documentation, SVM achieved comparable or better performance in most scenarios, especially in mixed-granularity and different-composition settings. Therefore, treeArches-SVM was used as the main treeArches setting in our benchmark.
